# Pre-Clovis occupation of the Americas identified by human fecal biomarkers in coprolites from Paisley Caves, Oregon

**DOI:** 10.1126/sciadv.aba6404

**Published:** 2020-07-15

**Authors:** Lisa-Marie Shillito, Helen L. Whelton, John C. Blong, Dennis L. Jenkins, Thomas J. Connolly, Ian D. Bull

**Affiliations:** 1 School of History, Classics and Archaeology, Armstrong Building, Newcastle University, Newcastle upon Tyne NE1 7RU, UK.; 2 Organic Geochemistry Unit, School of Chemistry, University of Bristol, Cantock’s Close, Bristol BS8 1TS, UK.; 3 Museum of Natural and Cultural History, University of Oregon, Eugene, OR 97403, USA.

## Abstract

When and how people first settled in the Americas is an ongoing area of research and debate. The earliest sites typically only contain lithic artifacts that cannot be directly dated. The lack of human skeletal remains in these early contexts means that alternative sources of evidence are needed. Coprolites, and the DNA contained within them, are one such source, but unresolved issues concerning ancient DNA taphonomy and potential for contamination make this approach problematic. Here, we use fecal lipid biomarkers to demonstrate unequivocally that three coprolites dated to pre-Clovis are human, raise questions over the reliance on DNA methods, and present a new radiocarbon date on basketry further supporting pre-Clovis human occupation.

## Teaser

We unequivocally confirmed pre-Clovis human occupation of the western Great Basin using fecal lipid biomarkers and a new 14C date.

## INTRODUCTION

It is largely, but not entirely, accepted by the archaeological community that people first settled the Americas before Clovis (*1*–*3*), which was seen as the earliest technological tradition on the continent for most of the 20th century, dating to 11,500 radiocarbon years before the present (^14^C yr B.P.) However, many questions still remain over who the earliest settlers were, when they arrived, and what route they took. The Western Stemmed Tradition (WST) is the oldest nonfluted lithic technology in the American Far West. Sites like Coopers Ferry, Idaho and Paisley Caves, Oregon provide evidence that WST technology predates Clovis, establishing this as the oldest well-defined technological tradition in North America, with links to late Pleistocene sites in East Asia and Siberia (*3*, *4*). The Paisley Caves contain additional evidence for pre-Clovis occupation in the form of coprolites dating to 12,400 ^14^C yr B.P. identified as human on the basis that they contain human DNA (*3*, *5*). This is a crucial case study as it remains the only site where a pre-Clovis WST assemblage has been found in direct association with well-preserved organic cultural material, enabling us to better understand these early settlers and their way of life.

Advances in sequencing and analyzing genomes of ancient people, plants, animals, and even microbiomes, has had a profound influence on the field of archaeology and our understanding of human evolution, but there are several criticisms of the technique, not least a lack of understanding of DNA taphonomy (*6*). DNA has been shown to be mobile in sediments in a range of environments and is also relatively easy to degrade. Because the polymerase chain reaction (PCR) method amplifies vanishingly low amounts of DNA, such analyses are particularly prone to contamination.

Because of the contentious nature of the first Americans debate and the uncertainties surrounding potential contamination, the findings from the coprolites from Paisley Caves have been the subject of criticism (*7*, *8*). Given the importance of the Paisley Caves, it is crucial to address outstanding questions about the age of the earliest deposits and reliability of ancient DNA (aDNA) analyses at the site. Currently, 285 radiocarbon dates have been obtained from Paisley Caves, and DNA analysis has been completed on 65 coprolites. The results presented by Jenkins *et al*. (*3*) showed that, in one sample, the dates obtained on macrofossils and water-soluble extracts from a Camelidae coprolite were 12,125 ± 30 ^14^C yr B.P. for the macrofossil and 11,315 ± 25 ^14^C yr B.P. for the water-soluble extract. This suggests that, in some samples, water-soluble compounds, including non-endogenous DNA, could be moving through the profile. Three coprolites identified as human were also dated this way but showed an agreement between the macroflora and solute dates. While this strongly supports the interpretation that humans occupied the site in pre-Clovis times, the ambiguity remains that DNA is potentially mobile in sediments (*9*, *10*), and linking a DNA signal with a specific radiocarbon date is problematic. Moreover, DNA is much more labile in the natural environment, susceptible to rapid degradation through hydrolytic cleavage of the phosphate ester backbone and oxidation of the pyrimidine bases and sugar moieties, although preservation through desiccation and association with minerals may enhance preservation (*11*).

**Fig. 1:**
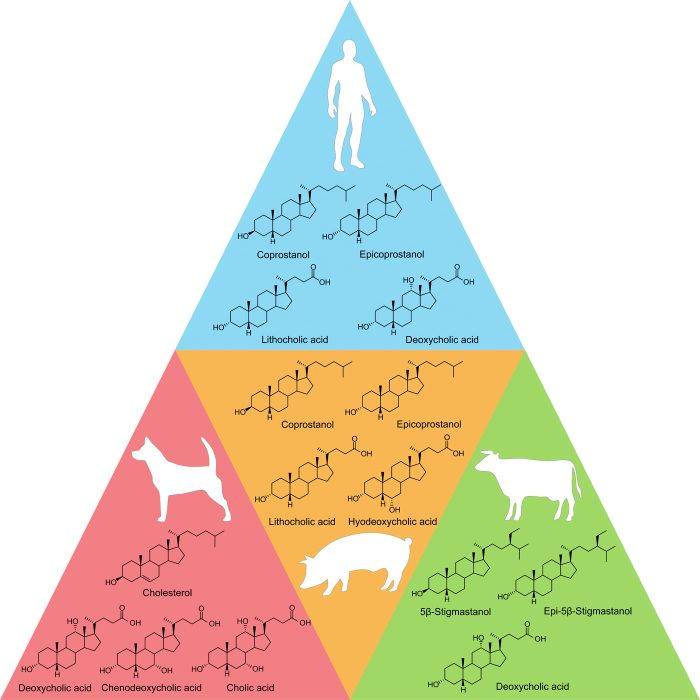
The dominant fecal sterol and bile acids in fecal material of humans, canids, pigs, and bovids.

As well as being repositories of DNA, coprolites also contain complex mixtures of lipids derived from the producer organism, its diet and biochemical processes occurring in its gut; some lipids are diagnostic of fecal origin [e.g., (*12*, *13*)]. In archaeology, lipid biomarkers have been used extensively in the analysis of ancient pottery, where they have revolutionized the study of ancient diet and other subsistence practices (*14*–*16*). Additional archaeological applications include agricultural soils [e.g., (*17*)] and human remains (*18*). Their innate hydrophobicity means that lateral and vertical movement within burial contexts is minimal (*19*, *20*). They are also chemically stable, with lipid biomarkers existing in rocks dating to 1.6 billion years (*21*), far greater than more recent archaeological time scales; 5β-stanols and bile acids are two lipid compound classes already well established as fecal biomarkers [see (*12*, *13*) and references therein]. Figure 1 summarizes how 5β-stanols and bile acids may be used to identify both fecal material and producer organism.

While not prevalent in archaeological investigations, fecal biomarkers have been successfully deployed, enabling the identification of a Roman latrine (*22*), identifying sanitation practices in ancient cities and early settlement sites (*23*–*25*), and identifying early wild animal penning and management before domestication (*26*). Critically, 5β-stanols and bile acids have a substantial advantage over DNA in that they are (i) hydrophobic and therefore unlikely to move within the stratigraphy of a sedimentary profile, (ii) highly recalcitrant (far more so than DNA) within a sedimentary context over archaeological time scales, and (iii) highly diagnostic of fecal material and its producer organism(s). Here, we present compelling new, fecal biomarker–based evidence that reveals, without any ambiguity from the interpretation, that pre-Clovis coprolites from the Paisley Caves complex are human.

## RESULTS

**Fig. 2:**
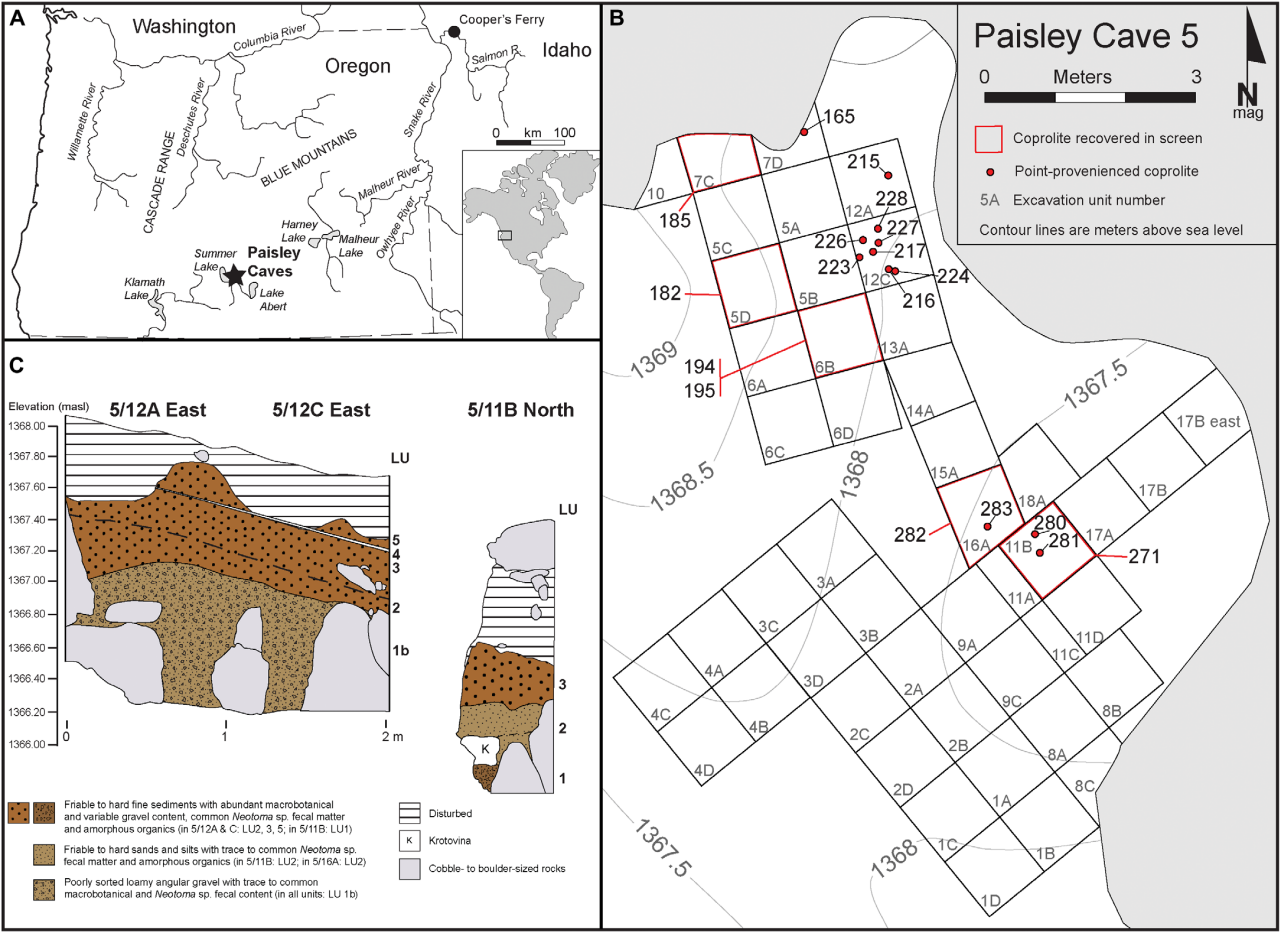
Site location map. (**A**) Location of Paisley Caves in the western Great basin. (**B**) Spatial location of coprolites in cave 5. (**C**) Stratigraphic profiles in cave 5.

**Fig. 3:**
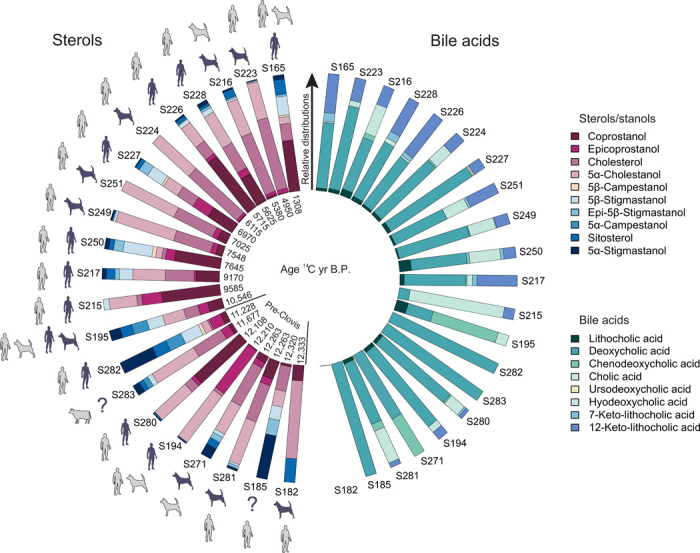
Summary of the fecal lipid biomarker and bile acid profiles of the coprolites analyzed, their species identified by DNA (light gray), their radiocarbon age, and determination of species (dark gray). Samples with multiple radiocarbon dates are combined using Oxcal 4.3 “R_combine” command.

**Fig. 4:**
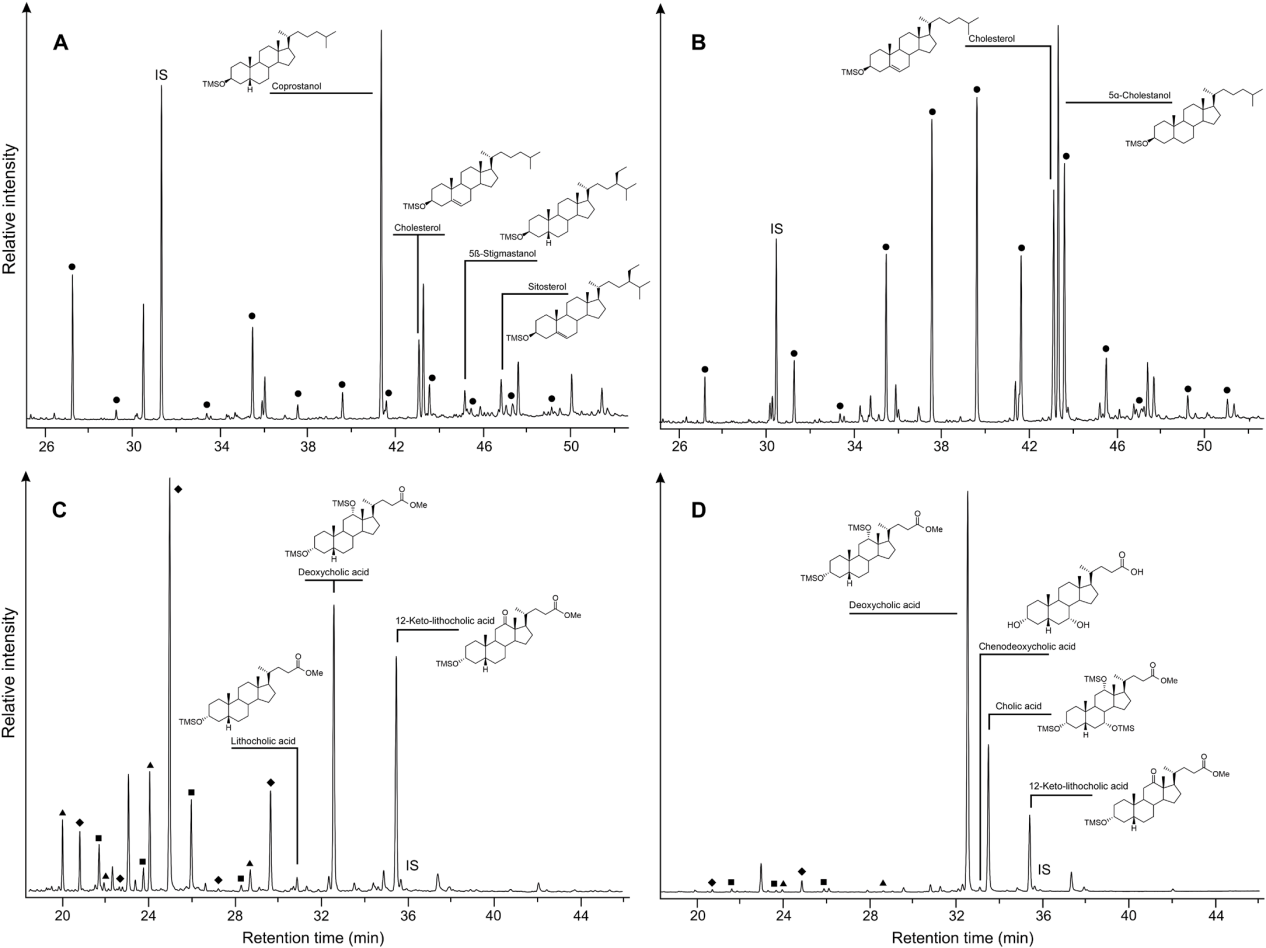
Partial gas chromatograms illustrating the distribution of steroid compounds in coprolites identified as human or carnivore. (**A**) human (S226) and (**B**) carnivore (S249), where ● denotes n-alcohols of carbon chain length C_20_ to C_32_, and their corresponding bile acids (**C**) human (S226) and (**D**) carnivore (S249), where ▲ denotes diacids (methylated), ■ denotes hydroxy fatty acid methyl esters (TMS derivatives), and ⧫ denotes ω-hydroxy fatty acid methyl esters (TMS derivatives). IS denotes added internal standards: Preg-5en-3β-ol is the sterol standard, and hyocholic acid is the standard for bile acids.

Twenty-one coprolites, previously subjected to mitochondrial DNA (mtDNA) analysis by Gilbert and coworkers (*3*, *5*), were subsampled, and their fecal biomarker (sterol and bile acid) content was determined (see Fig. 2 and fig. S1 for site and coprolite location and data S1 for physical descriptions). Figure 3 and data S1 summarize the results obtained from fecal biomarker analysis of the coprolites and provide an origin assignment based on criteria originally proposed by Bull and coworkers (*12*) (see fig. S2). In addition to criteria explicitly outlined in fig. S2, carnivore identifications were confirmed with a coprostanol to cholesterol ratio of <1 (see Table 1), in conjunction with a predominance of deoxycholic acid and a higher proportion of cholic acid in the bile acid profiles. Figure 4 shows examples of gas chromatography (GC) profiles illustrating the distribution of steroid compounds for coprolites identified as human (S226) and carnivore (S249). In addition, all but one of the coprolites has an associated radiocarbon date (see table S1). Two of the coprolites yielded inconclusive results, most likely arising from a mixture of environmentally derived lipids and low fecal lipid preservation. Of the 18 coprolites identified as containing human mtDNA by Gilbert and coworkers, 10 are interpreted as human in origin, 6 are ascribed a carnivore origin, and a remaining coprolite is most likely of mixed (human/carnivore) origin. Of the six pre-Clovis coprolites identified as human by mtDNA, three are confirmed as human by this analysis (Fig. 2 and Table 1), supporting the pre-Clovis age of the earliest occupation at Paisley Caves. Both carnivore mtDNA coprolites contain fecal biomarkers consistent with a carnivore origin.

**Table 1:** Classifications of coprolites and sediment sample based on the relative distributions of fecal biomarkers. LCA, lithocholic acid; DCA, deoxycholic acid; CDCA, chenodeoxycholic acid; CA, cholic acid; UDCA, ursodeoxycholic acid; 7-keto-LCA, 7-keto-lithocholic acid; 12-keto-LCA, 12-keto-lithocholic acid; n/a, not applicable; nd, not determined. Bile acids are presented in the order of abundance.

**Sample** **ID**	**Paisley** **catalog** **number**	**Published aDNA** **results (*3*, *5*)**	**Ratio 2** **(*12*)**	**Ratio 3** **(*12*)**	**Coprostanol/** **cholesterol**	**Bile acids**	**Lipid** **biomarker** **results**	**Age ^14^C yr B.P.**
165	1294-PC-5/7D- 4-3a	*H. sapiens* mtDNA haplogroup B2, *Canis latrans*	0.9	2.5	3.0	DCA, 12-keto-LCA, 7-keto-LCA, LCA, CA	Human	1308 ± 28 (OxA-16377)
223	1704-PC- 5/12C-13-6a	*H. sapiens* mtDNA haplogroup A	0.1	2.7	0	DCA, 12-keto-LCA LCA, CA	Carnivore	4950 ± 15 (UCIAMS-79710)
216	1704-PC- 5/12C-13-4a	*L. rufus*	0.1	1.5	0	DCA, CA, 12-keto-LCA CDCA, LCA	Carnivore	5380 ± 15 (UCIAMS-79715)
228	1704-PC- 5/12C-12-6a	*H. sapiens* mtDNA haplogroup A	0.5	5	1.1	DCA, 12-keto-LCA, 7-keto-LCA, LCA, CA.	Human	5595 ± 15 (UCIAMS-79702); 5655 ± 15 (UCIAMS-79703)
226	1704-PC- 5/12C-14-6a	*H. sapiens* mtDNA haplogroup A	0.7	13.6	4.0	DCA, 12-keto-LCA, 7-keto-LCA, LCA, CA	Human	5715 ± 15 (UCIAMS-76186)
224	1704-PC- 5/12C-15-5a	*H. sapiens* mtDNA haplogroup B	0.1	0	0	DCA, CA, 12-keto-LCA, LCA, CDCA	Carnivore	6115 ± 15 (UCIAMS-76185)
227	1704-PC- 5/12C-14-9a	*H. sapiens* mtDNA haplogroup A	0.7	1.8	1.5	DCA, 12-keto-LCA, LCA, CA, UDCA	Human	6970 ± 15 (UCIAMS-76180)
251	1830-PC- 2/4D-33-2	*H. sapiens* mtDNA haplogroup A	0.2	0	0.2	DCA, 12-keto-LCA, CA, 7-keto-LCA, LCA	Carnivore	7025 ± 15 (UCIAMS-79713)
249	1830-PC- 2/4C-34-101	*H. sapiens* mtDNA haplogroup A	0.2	3.8	0.2	DCA, CA, 12-keto-LCA, CDCA, LCA, 7-keto-LCA,	Carnivore	7490 ± 20 (UCIAMS-79704); 7605 ± 20 (UCIAMS-79705)
250	1830-PC- 2/4-D-33	*H. sapiens* mtDNA haplogroup A	0.9	1.4	12.4	DCA, CA, LCA, 12-keto- LCA, 7-keto-LCA	Human	7645 ± 20 (UCIAMS-79712)
217	1704-PC- 5/12A-16-9a	*H. sapiens* mtDNA haplogroup A	0.4	1.4	–	DCA, 12-keto-LCA, LCA, CA, 7-keto-LCA, CDCA	Human	9170 ± 20 (UCIAMS-76183)
215	1704-PC- 5/12A-16-10a	*H. sapiens* mtDNA haplogroup A	0.7	13.4	11.2	CA, DCA, 12-keto-LCA LCA	Human	9585 ± 20 (UCIAMS-76181)
195	1294-PC-5/6B- 40-6a	*H. sapiens* mtDNA haplogroup B2, *C. lupus/familiaris*	0.5	1.5	1.0	CDCA, DCA, CA, LCA	Human/ carnivore*	10,050 ± 50 (β-213423); 10,965 ± 50 (OxA-16376)
282	1896-PC- 5/16A-24-7	*H. sapiens* mtDNA haplogroup A	0.7	1.0	6.4	DCA, LCA	Human	11,205 ± 25 (UCIAMS-90583); 11,250 ± 25 (UCIAMS-90584)
283	1896-PC- 5/16A-25-12a	*Camelidae*	0.2	2.4	–	DCA	n/a	11,315 ± 25 (UCIAMS-90586); 12,125 ± 30 (UCIAMS-90589)
280	1830-PC- 5/11B-33	*H. sapiens* mtDNA haplogroup A	0.6	nd	6.9	DCA, CA, LCA, 12-keto- LCA, 7-keto-LCA, CDCA	Human	12,050 ± 25 (UCIAMS-79707); 12,165 ± 25 (UCIAMS-79706)
194	1294-PC-5/6B- 50-5a^†^	*H. sapiens* mtDNA haplogroup A2, *V. vulpes*	0.5	nd	32.2	DCA, LCA, 12-keto- LCA, CA	Human	12,140 ± 70 (OxA-16495); 12,260 ± 60 (β-216474)
271	1830-PC- 5/11B-31-12	*P. leo*	0.3	1.4	0.2	DCA, CDCA	Carnivore	From same context as coprolite 281.
281	1830-PC- 5/11B-31-2	*H. sapiens* mtDNA haplogroup B	0.4	5.7	0.5	DCA, CA, CDCA, 12-keto-LCA, LCA	Carnivore	12,260 ± 30 (UCIAMS-76190); 12,265 ± 25 (UCIAMS-76190)
185	1294-PC-5/7C- 31-9a	*H. sapiens* mtDNA haplogroup B2	0.1	0.1	0.1	DCA	n/a	12,290 ± 60 (β-213426); 12,345 ± 55 (OxA-16497)
182	1374-PC-5/5D- 31-2a^5^	*H. sapiens* mtDNA haplogroup B?	0.2	nd	–	nd	Carnivore^‡^	12,275 ± 55 (OxA-16498); 12,400 ± 60 (β-213424)

*Possible mixing of fecal matter due to high proportion of CDCA in bile acids.

†Several coprolites are associated with the same catalog number, and we cannot be 100% certain that we analyzed the same coprolite that was analyzed for mtDNA.

‡Potentially carnivore (unable to confirm due to lack of bile acids).

To further address outstanding questions about the age of the earliest deposits, a bulrush shaft with the features of an S-twist (clockwise) twined basket or mat weft fragment (fig. S3) was submitted for accelerator mass spectrometry (AMS) radiocarbon analysis. Twining, the only basketry type known from the region before the late Holocene, uses a pair of weft elements twisted around a warp in a clockwise (stitches oriented up to the right) or counterclockwise (down to the right) direction. The dated element has a slight clockwise twist and is crimped and flattened, where it would press against opposing warps. Bulrush is one of the most common basketry fibers in the region; it is not natural to the cave deposits and is a cultural addition. This artifact was recovered above coprolite 185 directly dated to 12,290 ± 60 and 12,345 ± 55 ^14^C yr B.P. (table S1) and yielded a radiocarbon date of 12,273 ± 56 ^14^C yr B.P. (table S3), confirming the pre-Clovis age and stratigraphic integrity of the deposit.

## DISCUSSION

### Burial context and preservation

All of the coprolites analyzed are identifiable as intact structures (figs. S4 and S5); this is critical as it constrains the interpretation of observed biomarkers to those consistent with the observed morphology, i.e., a species assignment of a rat (for example) would not be viable, as it would be inconsistent with the shape of the coprolite. Comparative assessment of lipids derived from coprolite 283 and a sediment sample collected directly underneath the coprolite (table S2) reveal distinctly different distributions where sediments are differentiated by a high level of *n*-alkanols (C_14_-C_34_) with an even-over-odd predominance, characteristic of an input from higher plants (*27*). Sterols, including sitosterol and stanols (5α-cholestanol and 5α-stigmastanol), microbially transformed in the environment, are also dominant within the lipid extract (*28*). Crucially, this shows that the lipids are representative of the matrix from which they are derived and have experienced minimal leaching or lateral movement within the sedimentary profile. Fecal biomarkers predominate in lipid extracts from coprolites recovered from the younger phases of the cave, and there is a general trend toward the concentration of fecal sterols decreasing as radiocarbon age of the coprolite increases from 573 μg g^−1^ in the youngest coprolite to 9.5 μg g^−1^ in the oldest coprolite. Lipids (and hence fecal biomarkers) are chemically stable and, although some microbial or diagenetic alterations do occur with time, the resulting structures can be linked back to their original source (*29*). Environmental conditions can greatly influence the preservation of fecal sterols (*30*–*33*). The slow deposition of sediment and aerobic conditions of the burial environment is likely the cause for the greater degradation of lipids in the oldest coprolites.

### Fecal biomarker results and coprolite origins

Fecal biomarker interpretations for 13 of 21 coprolites analyzed agree with mtDNA classifications reported previously (Table 1) (*3*, *5*). Of these, the majority indicate a human origin (both *Homo sapiens* mtDNA haplogroups A and B) and range in age from 1308 ± 28 to 12,260 ± 60 ^14^C yr B.P. Two of the 13 coprolites reported as containing mtDNA from *Lynx rufus* (216) and *Panthera leo* (271) yielded fecal biomarker distributions consistent with those of a carnivore. A single coprolite (195) reported to contain mtDNA from *H. sapiens* and *Canis lupus*/*familiaris* also yielded fecal biomarker distributions indicative of a mixed human/carnivore origin. One explanation for these results from a single specimen is coprophagy of human feces by carnivores. Coprophagy in canids is a well-recognized phenomenon (*34*) and could explain both the presence of carnivore-derived lipids and human mtDNA, but it is unlikely that humans were eating carnivores as suggested by Gilbert and coworkers (*6*). This is supported by the fecal biomarker evidence, i.e., only coprophagy would result in a human/canid mix of biomarkers in a discrete coprolite. Moreover, not only carnivores have a lower biomass transfer efficiency but also consumption of carnivore meat can be hazardous to human health due to parasitic infections and the bioaccumulation of toxins (*35*–*37*). Of the remaining eight coprolites, two did not yield enough lipid to be viable for biomarker analysis. At 12,125 ± 30 ^14^C yr B.P. (283) and 12,345 ± 55 ^14^C yr B.P. (185), these both represent coprolites from the oldest deposits in the cave and a lower recovery of lipid is therefore not unexpected.

Six of the coprolites classified as human using mtDNA contain fecal biomarkers indicative of carnivores. Sterols derived from canid feces are characterized by very high levels of cholesterol, and only trace amounts (if any) of cholesterol-derived 5β-stanols (coprostanol and epicoprostanol) as canids lack the gut microbiota responsible for producing these compounds (*38*). Carnivores differ from other animals in their bile acid composition and are identifiable from the higher levels of cholic and chenodeoxycholic acids in addition to deoxycholic acid (*39*–*42*). Conversely, human feces are usually characterized by high levels of coprostanol and higher amounts of deoxycholic acid and lithocholic acid (*43*). Previous work has reported that about a quarter of North American subjects in a study exhibited little to no conversion of cholesterol to coprostanol (*44*). This may potentially result in false negatives when differentiating human coprolites from carnivores and may explain the lack of agreement between mtDNA and fecal biomarker results in these six coprolites.

### Implications for aDNA analysis and early human occupation

mtDNA analyses rely upon amplification of ultratrace amounts of extant mtDNA using the PCR, thereby making such an approach not only more sensitive but also more susceptible to contamination (*45*). Conversely, fecal biomarker analyses are reliant upon coprolites containing enough of each compound to enable detection and quantification (typically in the region of >0.4 μg g^−1^_coprolite_), with the concentration of compounds remaining being a direct reflection of age and depositional environment (*30*–*33*). However, because of their source specificity, contamination is very unlikely. Ideally, both techniques should be used together in a complementary fashion for the assessment of feces from both single and multiple sources. While fecal biomarker analysis lacks the detailed information that mtDNA may provide, it does deliver a more rapid assessment of coprolites that, as in this study, can provide robust supporting evidence where results obtained by mtDNA-based approaches are open to criticism. It also represents an ideal screening method before attempting mtDNA analyses that are far more laborious and require a greater degree of verification, i.e., Gilbert and coworkers (*6*) used several different DNA typing and sequencing methods across six independent laboratories. Where no fecal biomarker results are available, the matrix either is of a nonfecal origin or is sufficiently old and/or has been environmentally exposed such that interpretations of a fecal origin using mtDNA only should be, at best, speculative.

The peopling of the Americas is a complex story. The continents are large, and early populations were likely to be sparsely distributed. The lack of early human skeletal remains, and the ethical issues associated with destructive analysis of the handful that have been discovered (*46*, *47*), means that coprolites are the best direct archaeological evidence that people occupied a particular site. Human occupation at Paisley Caves is now proven to 12,200 ^14^C yr B.P. using fecal lipid biomarkers. Coprolites 194 and 280 are the oldest coprolites determined to be unequivocally human based on fecal biomarker analysis, a methodology that bypasses current uncertainties surrounding mtDNA. These coprolites also show agreement between both mtDNA and fecal biomarker analyses. Fecal lipid biomarkers offer access to hitherto unknown (or at best, uncertain) information, adding to the growing body of evidence that is helping to build a picture of not only when and by what route people arrived but also how these people adapted to diverse landscapes across the continent (*48*–*50*).

## MATERIALS AND METHODS

Where possible, the exterior of the coprolite was removed with a scalpel before analysis to minimize any environmental contamination. Subsequent to solvent extraction and compound class isolation, the coprolites were analyzed by GC and GC-MS to characterize potentially extant organic molecules.

### Extraction of lipids from coprolites

Approximately 0.5 g of a coprolite was crushed using a mortar and pestle and then passed through a 2-mm sieve. Suitable amounts of two internal standards (hyocholic acid and preg-5-en-3β-ol; 50 μl, 0.1 mg ml^−1^ solution) were added to the powdered samples. The lipids were then microwave-extracted into 10 ml of 2:1 dichloromethane/CH_3_OH (v/v). The total lipid extract obtained was subsequently hydrolyzed, and the sterol and bile acid fractions were isolated as outlined by Bull *et al*. (*51*) and Elhmmali *et al*. (*43*), respectively. For the methylation of bile acids, BF_3_-methanol was used as an alternative methylation reagent. The fractions containing the target biomarkers were then analyzed by GC and GC-MS.

### Instrumental analyses

Initial screening of biomarker isolates was performed using an HP 5890 Series II gas chromatograph. Trimethylsilylated sterols and methylated and trimethylsilylated bile acids were introduced (1.0 μl) via an on-column injector. The analytical column was a 50 m × 0.32 mm fused silica capillary column coated with a 100% dimethylpolysiloxane nonpolar stationary phase (HP-1, 0.17 μm; Agilent). For the sterol analyses, the GC temperature program was set to hold at 50°C for 2 min, followed by a gradient increase to 200°C at 10°C min^−1^, and then to 300°C at 3°C min^−1^ before a final isothermal at 300°C for 20 min. For the analyses of bile acids, the GC temperature program was set to hold at 40°C for 1 min, followed by a gradient increase to 230°C at 20°C min^−1^, and then to 300°C at 2°C min^−1^ before a final isothermal at 300°C for 20 min. Helium was used as the carrier gas set to constant flow of 2.0 ml min^−1^, and the flame ionization detector (FID) used to monitor column effluent was kept at a constant temperature of 300°C. Data were acquired using DataApex Clarity (version 2.6.2.226).

GC-MS analyses of sterols and bile acids were performed using an Agilent 7890-7200B GC/Q-TOFMS. Samples (1.0 μl) were injected using a 7693 autosampler and a multimode inlet (set to track the oven temperature) onto a 50 m × 0.32 mm (Agilent) fused silica capillary column coated with a 100% dimethylpolysiloxane nonpolar stationary phase (HP-1, 0.17 μm; Agilent). The GC temperature programs used were the same as for the corresponding GC analyses. Helium was used as the carrier gas, set to a constant flow of 2 ml min^−1^. The GC transfer line, ion source, and quadrupole were set to 320, 230, and 150°C, respectively. The MS was set to acquire in the range of *m/z* (mass to charge ratio) 50 to 1050 at a scan rate of 0.2 Hz in extended dynamic range mode.

## Supplementary Material

aba6404_SM.pdf

aba6404_Data_S1.xlsx

## SUPPLEMENTARY MATERIALS

Supplementary material for this article is available at http://advances.sciencemag.org/cgi/content/full/6/29/eaba6404/DC1

## References

[1] Wheat A., Survey of professional opinions regarding the peopling of the Americas. SAA Archaeol. Rec. 12, 10–14 (2012).

[2] Braje T. J., Dillehay T. D., Erlandson J. M., Klein R. G., Rick T. C., Finding the first Americans. Science 358, 592–594 (2017). 29097536 10.1126/science.aao5473

[3] Jenkins D. L., Davis L. G., Stafford T. W. Jr., Campos P. F., Hockett B., Jones G. T., Cummings L. S., Yost C., Connolly T. J., Yohe R. M. II, Gibbons S. C., Raghavan M., Rasmussen M., Paijmans J. L. A., Hofreiter M., Kemp B. M., Barta J. L., Monroe C., Gilbert M. T. P., Willerslev E., Clovis age Western Stemmed projectile points and human coprolites at the Paisley Caves. Science 337, 223–228 (2012). 22798611 10.1126/science.1218443

[4] Davis L. G., Madsen D. B., Becerra-Valdivia L., Higham T., Sisson D. A., Skinner S. M., Stueber D., Nyers A. J., Keen-Zebert A., Neudorf C., Cheyney M., Izuho M., Iizuka F., Burns S. R., Epps C. W., Willis S. C., Buvit I., Late Upper Paleolithic occupation at Cooper’s Ferry, Idaho, USA, ~16,000 years ago. Science 365, 891–897 (2019). 31467216 10.1126/science.aax9830

[5] Gilbert M. T. P., Jenkins D. L., Götherstrom A., Naveran N., Sanchez J. J., Hofreiter M., Thomsen P. F., Binladen J., Higham T. F. G., Yohe R. M. II, Parr R., Cummings L. S., Willerslev E., DNA from pre-Clovis human coprolites in Oregon, North America. Science 320, 786–789 (2008). 18388261 10.1126/science.1154116

[6] Pedersen M. W., Overballe-Petersen S., Ermini L., Sarkissian C. D., Haile J., Hellstrom M., Spens J., Thomsen P. F., Bohmann K., Cappellini E., Schnell I. B., Wales N. A., Carøe C., Campos P. F., Schmidt A. M. Z., Gilbert M. T. P., Hansen A. J., Orlando L., Willerslev E., Ancient and modern environmental DNA. Philos. Trans. R. Soc. Lond. B Biol. Sci. 370, 20130383 (2015). 25487334 10.1098/rstb.2013.0383 PMC4275890

[7] Poinar H., Fiedel S., King C. E., Devault A. M., Bos K., Kuch M., Debruyne R., Comment on “DNA from pre-Clovis human coprolites in Oregon, North America”. Science 325, 148 (2009). 10.1126/science.1168182 19589985

[8] Goldberg P., Berna F., Macphail R. I., Comment on “DNA from pre-Clovis human coprolites in Oregon, North America”. Science 325, 148 (2009). 10.1126/science.1167531 19589984

[9] Haile J., Holdaway R., Oliver K., Bunce M., Gilbert M. T. P., Nielsen R., Munch K., Ho S. Y. W., Shapiro B., Willerslev E., Ancient DNA chronology within sediment deposits: Are paleobiological reconstructions possible and is DNA leaching a factor? Mol. Biol. Evol. 24, 982–989 (2007). 17255121 10.1093/molbev/msm016

[10] Andersen K., Bird K. L., Rasmussen M., Haile J., Breuning-Madsen H., Kjaer K. H., Orlando L., Gilbert M. T. P., Willerslev E., Meta-barcoding of 'dirt' DNA from soil reflects vertebrate biodiversity. Mol. Ecol. 21, 1966–1979 (2012). 21917035 10.1111/j.1365-294X.2011.05261.x

[11] Romanowski G., Lorenz M. G., Wackernagel W., Adsorption of plasmid DNA to mineral surfaces and protection against DNase I. Appl. Environ. Microbiol. 57, 1057–1061 (1991). 1647748 10.1128/aem.57.4.1057-1061.1991 PMC182845

[12] Bull I. D., Lockheart M. J., Elhmmali M. M., Roberts D. J., Evershed R. P., The origin of faeces by means of biomarker detection. Environ. Int. 27, 647–654 (2002). 11934114 10.1016/s0160-4120(01)00124-6

[13] Prost K., Birk J. J., Lehndorff E., Gerlach R., Amelung W., Steroid biomarkers revisited - improved source identification of faecal remains in archaeological soil material. PLOS ONE 12, e0164882 (2017). 28060808 10.1371/journal.pone.0164882 PMC5217961

[14] Dunne J., Evershed R. P., Salque M., Cramp L., Bruni S., Ryan K., Biagetti S., di Lernia S., First dairying in green Saharan Africa in the fifth millennium BC. Nature 486, 390–394 (2012). 22722200 10.1038/nature11186

[15] Cramp L. J. E., Jones J., Sheridan A., Smyth J., Whelton H., Mulville J., Sharples N., Evershed R. P., Immediate replacement of fishing with dairying by the earliest farmers of the Northeast Atlantic archipelagos. Proc. Biol. Sci. 281, 20132372 (2014). 24523264 10.1098/rspb.2013.2372 PMC4027381

[16] Roffet-Salque M., Regert M., Evershed R. P., Outram A. K., Cramp L. J. E., Decavallas O., Dunne J., Gerbault P., Mileto S., Mirabaud S., Pääkkönen M., Smyth J., Šoberl L., Whelton H. L., Alday-Ruiz A., Asplund H., Bartkowiak M., Bayer-Niemeier E., Belhouchet L., Bernardini F., Budja M., Cooney G., Cubas M., Danaher E. M., Diniz M., Domboróczki L., Fabbri C., González-Urquijo J. E., Guilaine J., Hachi S., Hartwell B. N., Hofmann D., Hohle I., Ibáñez J. J., Karul N., Kherbouche F., Kiely J., Kotsakis K., Lueth F., Mallory J. P., Manen C., Marciniak A., Maurice-Chabard B., McGonigle M. A., Mulazzani S., Özdoğan M., Perić O. S., Perić S. R., Petrasch J., Pétrequin A.-M., Pétrequin P., Poensgen U., Pollard C. J., Poplin F., Radi G., Stadler P., Stäuble H., Tasić N., Urem-Kotsou D., Vuković J. B., Walsh F., Whittle A., Wolfram S., Zapata-Peña L., Zoughlami J., Widespread exploitation of the honeybee by early Neolithic farmers. Nature 527, 226–230 (2015). 26560301 10.1038/nature15757

[17] Bull I. D., Evershed R. P., Betancourt P. P., An organic geochemical investigation of the practice of manuring at a Minoan site on Pseira Island, Crete. Geoarchaeology 16, 223–242 (2001).

[18] Clark K. A., Ikram S., Evershed R. P., Organic chemistry of balms used in the preparation of pharaonic meat mummies. Proc. Natl. Acad. Sci. U.S.A. 110, 20392–20395 (2013). 24248384 10.1073/pnas.1315160110 PMC3870666

[19] R. P. Evershed, P. H. Bethell, in *Archaeological Chemsitry, Organic, Inorganic and Biochemical Analysis*, M. V. Orna, Ed., ACS Symposium Series, 625, Chapter 13 (American Chemical Society, Washington, DC, USA, 1996), 459 p.

[20] Lloyd C. E. M., Michaelides K., Chadwick D. R., Dungait J. A. J., Evershed R. P., Tracing the flow-driven vertical transport of livestock-derived organic matter through soil using biomarkers. Org. Geochem. 43, 56–66 (2012).

[21] Brocks J. J., Love G. D., Summons R. E., Knoll A. H., Logan G. A., Bowden S. A., Biomarker evidence for green and purple sulphur bacteria in a stratified Palaeoproterozoic sea. Nature 437, 866–870 (2005). 16208367 10.1038/nature04068

[22] Knights B. A., Dickson C. A., Dickson J. H., Breeze D. J., Evidence concerning the Roman military diet at Bearsden, Scotland, in the 2nd century AD. J. Archaeol. Sci. 10, 139–152 (1983).

[23] Bull I. D., Elhmmali M. M., Roberts D. J., Evershed R. P., The application of steroidal biomarkers to track the abandonment of a Roman wastewater course at the Agora (Athens, Greece). Archaeometry 45, 149–161 (2003).

[24] Shillito L.-M., Bull I. D., Matthews W., Almond M. J., Williams J. M., Evershed R. P., Biomolecular and micromorphological analysis of suspected faecal deposits at Neolithic Çatalhöyük, Turkey. J. Archaeol. Sci. 38, 1869–1877 (2011).

[25] Ledger M. L., Grimshaw E., Fairey M., Whelton H. L., Bull I. D., Ballantyne R., Knight M., Mitchell P. D., Intestinal parasites at the late bronze age settlement of must farm, in the fens of East Anglia, UK (9th century B.C.E.). Parasitology 146, 1583–1594 (2019). 31391134 10.1017/S0031182019001021

[26] L. M. Shillito, W. Matthews, I. D. Bull, J. Williams, in *The Earliest Neoltihic of Iran: 2008 Excavations at Sheik-E Abad and Jani*, R. Matthews, W. Matthews, Y. Mohammadifar, Eds. (Oxbow Books, 2013).

[27] Eglinton G., Hamilton R. J., Leaf epicuticular waxes. Science 156, 1322–1335 (1967). 4975474 10.1126/science.156.3780.1322

[28] Gaskell S. J., Eglinton G., Rapid hydrogenation of sterols in a contemporary lacustrine sediment. Nature 254, 209–211 (1975).

[29] Eglinton G., Logan G. A., Ambler R. P., Boon J. J., Perizonius W. R. K., Molecular preservation [and Discussion]. Philos. Trans. R. Soc. B 333, 315–328 (1991). 10.1098/rstb.1991.0081 1684047

[30] Nishimura M., Koyama T., The occurrence of stanols in various living organisms and the behavior of sterols in contemporary sediments. Geochim. Cosmochim. Acta 41, 379–385 (1977).

[31] Hatcher P. G., McGillivary P. A., Sewage contamination in the New York Bight. Coprostanol as an indicator. Environ. Sci. Technol. 13, 1225–1229 (1979).

[32] McCalley D. V., Cooke M., Nickless G., Coprostanol in Severn Estuary sediments. Bull. Environ. Contam. Toxicol. 25, 374–381 (1980). 6775719 10.1007/BF01985541

[33] Bartlett P. D., Degradation of coprostanol in an experimental system. Mar. Pollut. Bull. 18, 27–29 (1987).

[34] Livingston T. R., Gipson P. S., Ballard W. B., Sanchez D. M., Krausman P. R., Scat removal: A source of bias in feces-related studies. Wildl. Soc. Bull. 33, 172–178 (2005).

[35] McGrew A. K., Ballweber L. R., Moses S. K., Stricker C. A., Beckmen K. B., Salman M. D., O’Hara T. M., Mercury in gray wolves (*Canis lupus*) in Alaska: Increased exposure through consumption of marine prey. Sci. Total Environ. 468-469, 609–613 (2014). 24056451 10.1016/j.scitotenv.2013.08.045 PMC3870183

[36] Schurer J. M., Pawlik M., Huber A., Elkin B., Cluff H. D., Pongracz J. D., Gesy K., Wagner B., Dixon B., Merks H., Bal M. S., Jenkins E. J., Intestinal parasites of gray wolves (*Canis lupus*) in northern and western Canada. Can. J. Zool. 94, 643–650 (2016).

[37] Moleon M., Martínez-Carrasco C., Muellerklein O. C., Getz W. M., Muñoz-Lozano C., Sánchez-Zapata J. A., Carnivore carcasses are avoided by carnivores. J. Anim. Ecol. 86, 1179–1191 (2017). 28609555 10.1111/1365-2656.12714

[38] Leeming R., Ball A., Ashbolt N., Nichols P., Using faecal sterols from humans and animals to distinguish faecal pollution in receiving waters. Water Res. 30, 2893–2900 (1996).

[39] Wildgrube H. J., Stockhausen H., Petri J., Füssel U., Lauer H., Naturally occurring conjugated bile acids, measured by high-performance liquid chromatography, in human, dog, and rabbit bile. J. Chromatogr. 353, 207–213 (1986). 3700515 10.1016/s0021-9673(01)87090-4

[40] Fernández G. J., Corley J. C., Capurro A. F., Identification of cougar and jaguar feces through bile acid chromatography. J. Wildl. Manag. 61, 506–510 (1997).

[41] Major M., Johnson M. K., Davis W. S., Kellogg T. F., Identifying scats by recovery of bile-acids. J. Wildl. Manag. 44, 290–293 (1980).

[42] Johnson M. K., Belden R. C., Aldred D. R., Differentiating mountain lion and bobcat scats. J. Wildl. Manag. 48, 239–244 (1984).

[43] Elhmmali M. M., Roberts D. J., Evershed R. P., Bile acids as a new class of sewage pollution indicator. Environ. Sci. Technol. 31, 3663–3668 (1997).

[44] Wilkins T. D., Hackman A. S., Two patterns of neutral steroid conversion in the feces of normal north americans. Cancer Res. 34, 2550–2554 (1974). 4843533

[45] Llamas B., Valverde G., Fehren-Schmitz L., Weyrich L. S., Cooper A., Haak W., From the field to the laboratory: Controlling DNA contamination in human ancient DNA research in the high-throughput sequencing era. Sci. Technol. Archaeol. Res. 3, 1–14 (2017).

[46] Callaway E., Ancient genome stirs ethics debate. Nature 506, 142–143 (2014). 24522580 10.1038/506142a

[47] Rasmussen M., Anzick S. L., Waters M. R., Skoglund P., De Giorgio M., Stafford T. W. Jr., Rasmussen S., Moltke I., Albrechtsen A., Doyle S. M., Poznik G. D., Gudmundsdottir V., Yadav R., Malaspinas A.-S., White S. S. V, Allentoft M. E., Cornejo O. E., Tambets K., Eriksson A., Heintzman P. D., Karmin M., Korneliussen T. S., Meltzer D. J., Pierre T. L., Stenderup J., Saag L., Warmuth V. M., Lopes M. C., Malhi R. S., Brunak S., Sicheritz-Ponten T., Barnes I., Collins M., Orlando L., Balloux F., Manica A., Gupta R., Metspalu M., Bustamante C. D., Jakobsson M., Nielsen R., Willerslev E., The genome of a Late Pleistocene human from a Clovis burial site in western Montana. Nature 506, 225–229 (2014). 24522598 10.1038/nature13025 PMC4878442

[48] Waters M. R., Forman S. L., Jennings T. A., Nordt L. C., Driese S. G., Feinberg J. M., Keene J. L., Halligan J., Lindquist A., Pierson J., Hallmark C. T., Collins M. B., Wiederhold J. E., The Buttermilk Creek complex and the origins of Clovis at the Debra L. Friedkin site, Texas. Science 331, 1599–1603 (2011). 21436451 10.1126/science.1201855

[49] Halligan J. J., Waters M. R., Perrotti A., Owens I. J., Feinberg J. M., Bourne M. D., Fenerty B., Winsborough B., Carlson D., Fisher D. C., Stafford T. W. Jr., Dunbar J. S., Pre-Clovis occupation 14,550 years ago at the Page-Ladson site, Florida, and the peopling of the Americas. Sci. Adv. 2, e1600375 (2016). 27386553 10.1126/sciadv.1600375 PMC4928949

[50] Goebel T., Waters M. R., O'Rourke D. H., The late Pleistocene dispersal of modern humans in the Americas. Science 319, 1497–1502 (2008). 18339930 10.1126/science.1153569

[51] Bull I. D., Simpson I. A., Dockrill S. J., Evershed R. P., Organic geochemical evidence for the origin of ancient anthropogenic soil deposits at Tofts Ness, Sanday, Orkney. Org. Geochem. 30, 535–556 (1999).

