# Supplementary material for: Pre-Clovis occupation of the Americas identified by human fecal biomarkers in coprolites from Paisley Caves, Oregon
Source: Sci Adv. 2020 Jul 15;6(29):eaba6404. doi: 10.1126/sciadv.aba6404 (PMC7363456; doi:10.1126/sciadv.aba6404)
Supplement: aba6404_SM.pdf [file aba6404_SM.pdf]

Science Advances
AAAS

advances.sciencemag.org/cgi/content/full/6/29/eaba6404/DC1

Supplementary Materials for

Pre-Clovis occupation of the Americas identified by human fecal biomarkers in coprolites from Paisley Caves, Oregon

Lisa-Marie Shillito*, Helen L. Whelton, John C. Blong, Dennis L. Jenkins, Thomas J. Connolly, Ian D. Bull*

*Corresponding author. Email: lisa-marie.shillito@newcastle.ac.uk (L.-M.S.); ian.d.bull@bristol.ac.uk (I.D.B.)

Published 15 July 2020, *Sci. Adv.* **6**, eaba6404 (2020)
DOI: 10.1126/sciadv.aba6404

The PDF file includes:

Figs. S1 to S5
Tables S1 and S2
Legend for data S1

Other Supplementary Material for this manuscript includes the following:

(available at advances.sciencemag.org/cgi/content/full/6/29/eaba6404/DC1)

Data S1

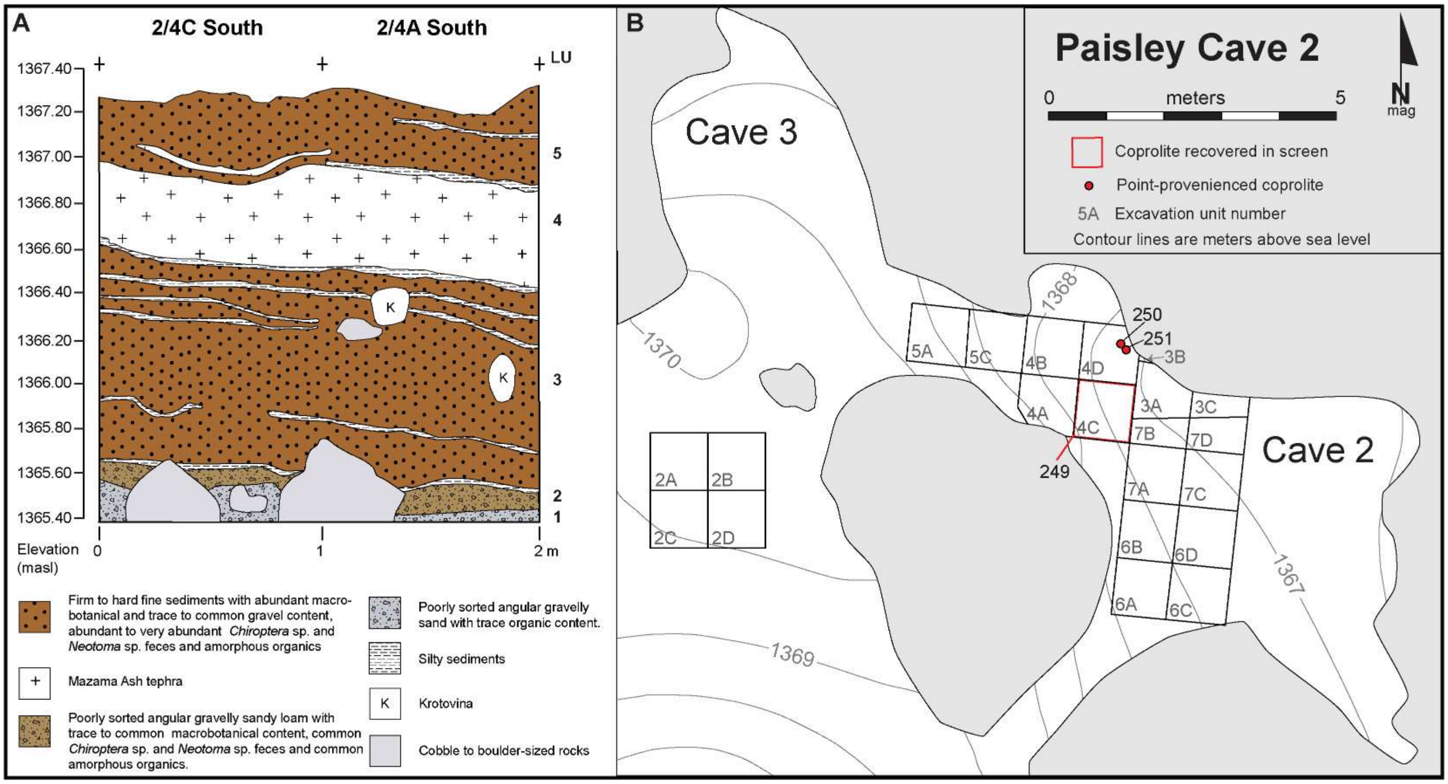

Fig. S1.

A, stratigraphic profile in cave 2; and B, the spatial location of coprolites in cave 2. All figures redrawn from (*3*).

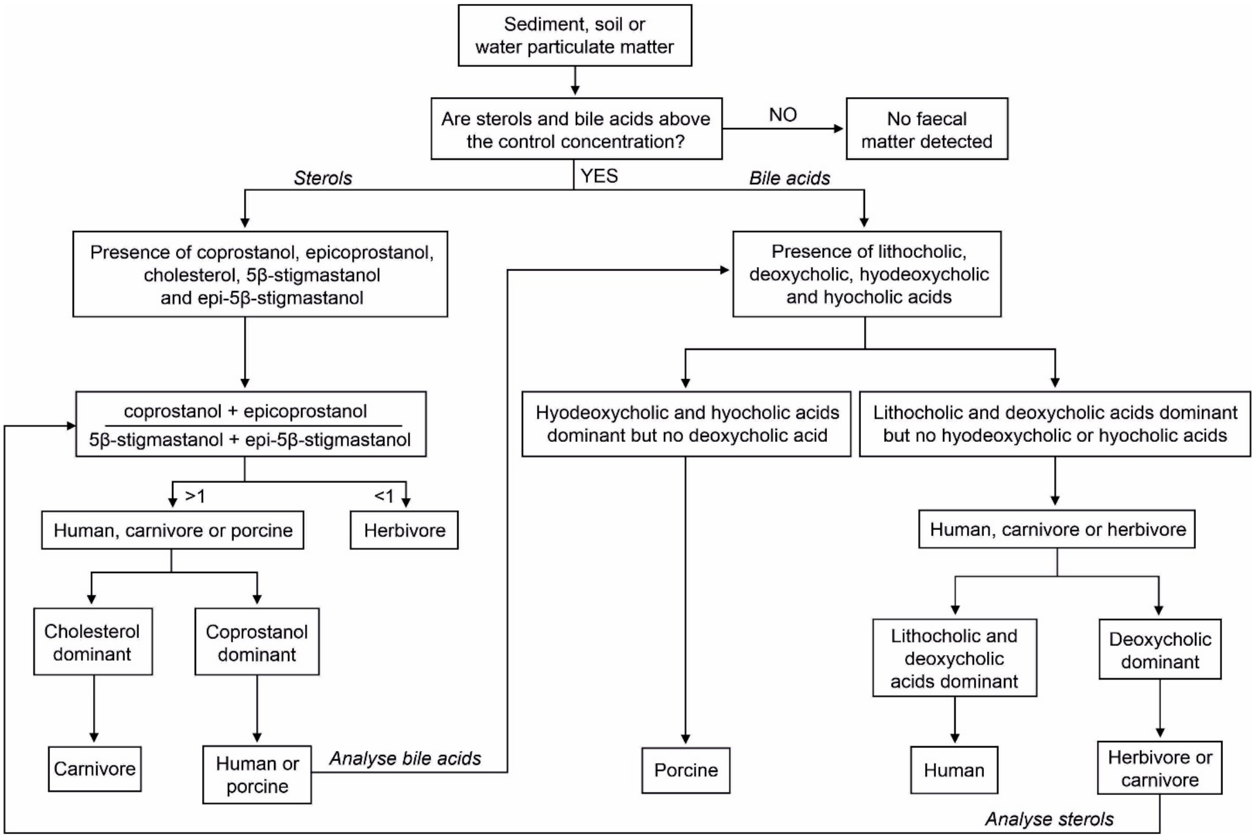

Fig. S2.

Flowchart outlining the criteria used to determine the source of faeces/faecal pollution using lipid biomarkers (adapted from *12*).

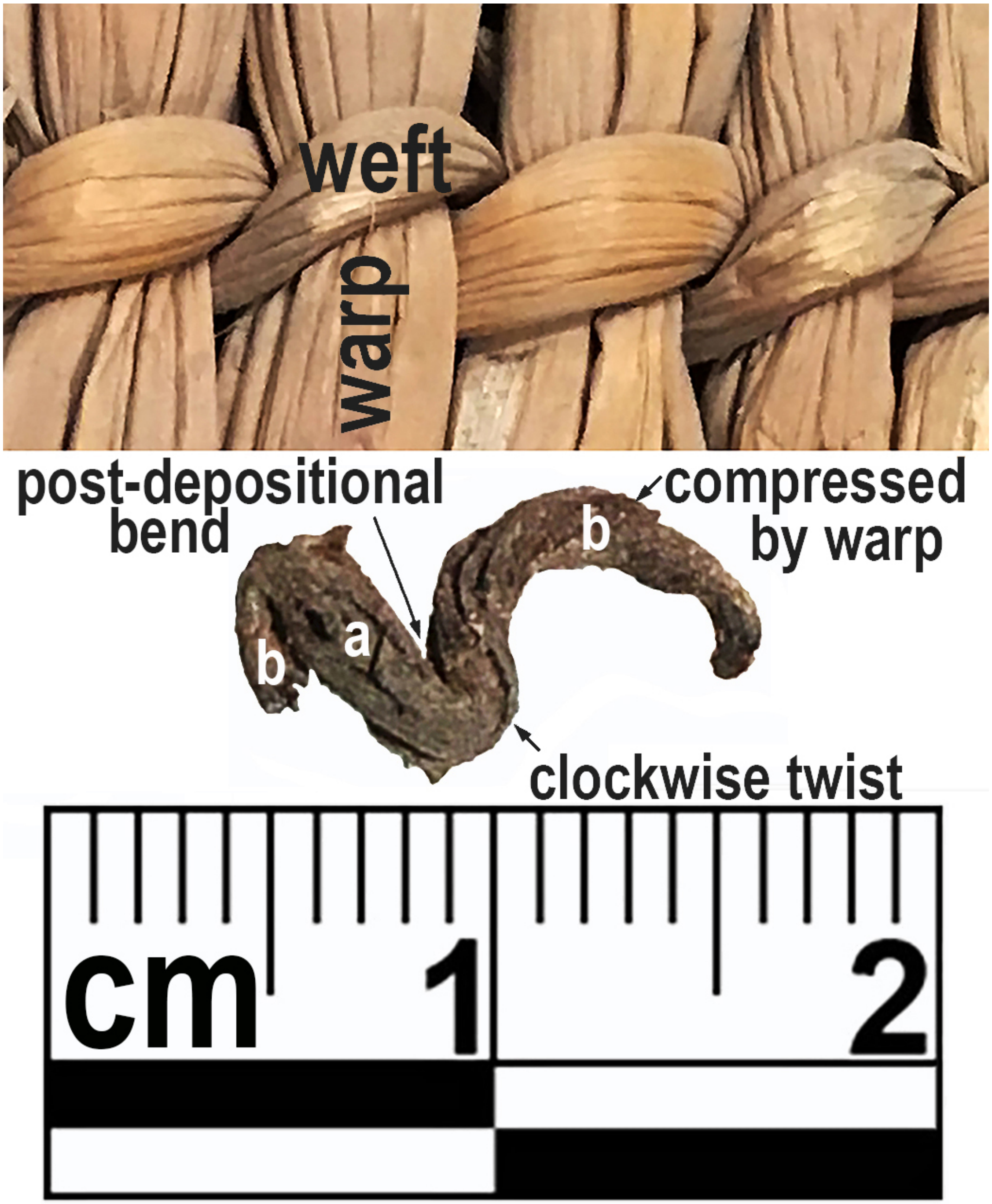

Fig. S3.

Lower image is the bulrush shaft with the features of an S-twist (clockwise) twined basket or mat weft fragment, dated to 12,273 ± 56 14C yr B.P. Upper image is a comparable archaeological example showing a twined weft row. The visible face of a weft is labelled "a" and the opposing face (hidden behind the warp) is labelled "b". Photo Credit: Thomas Connolly. University of Oregon.

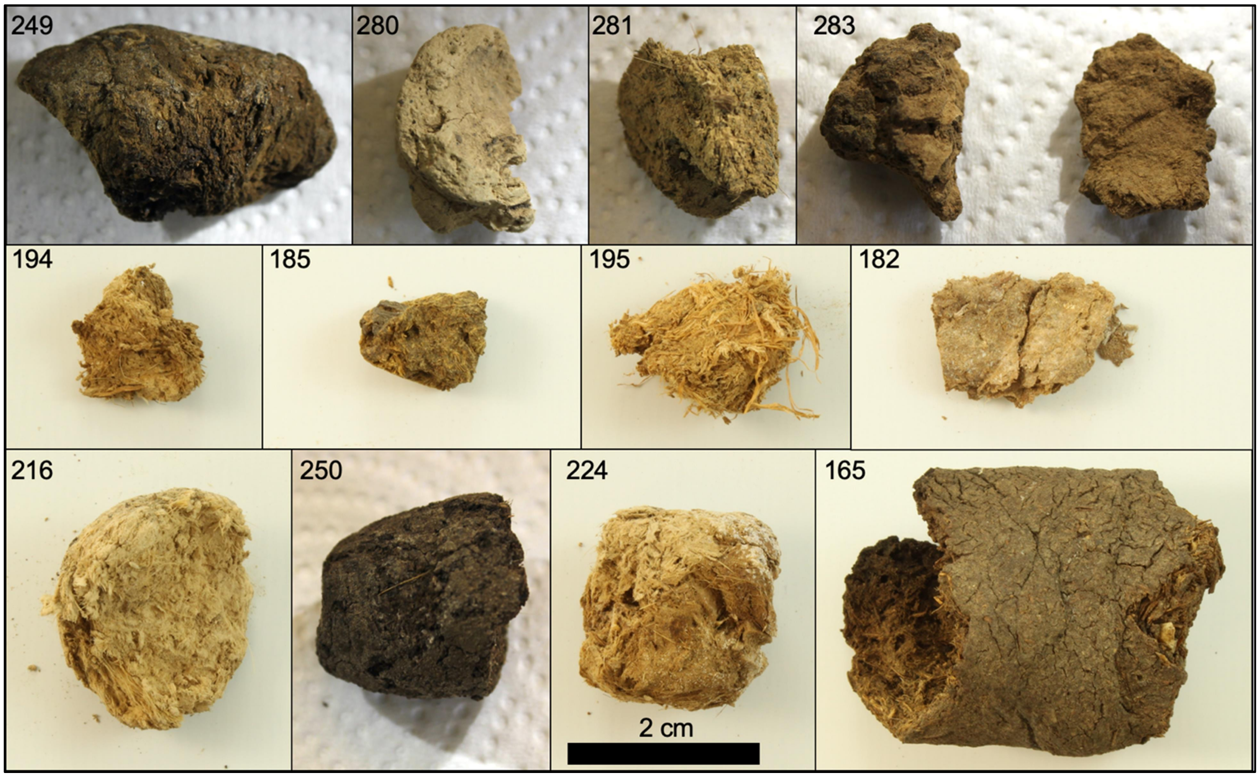

Fig. S4.

Figure S4. Images of Paisley coprolites prior to subsampling. Photo Credit: John Blong, Newcastle University.

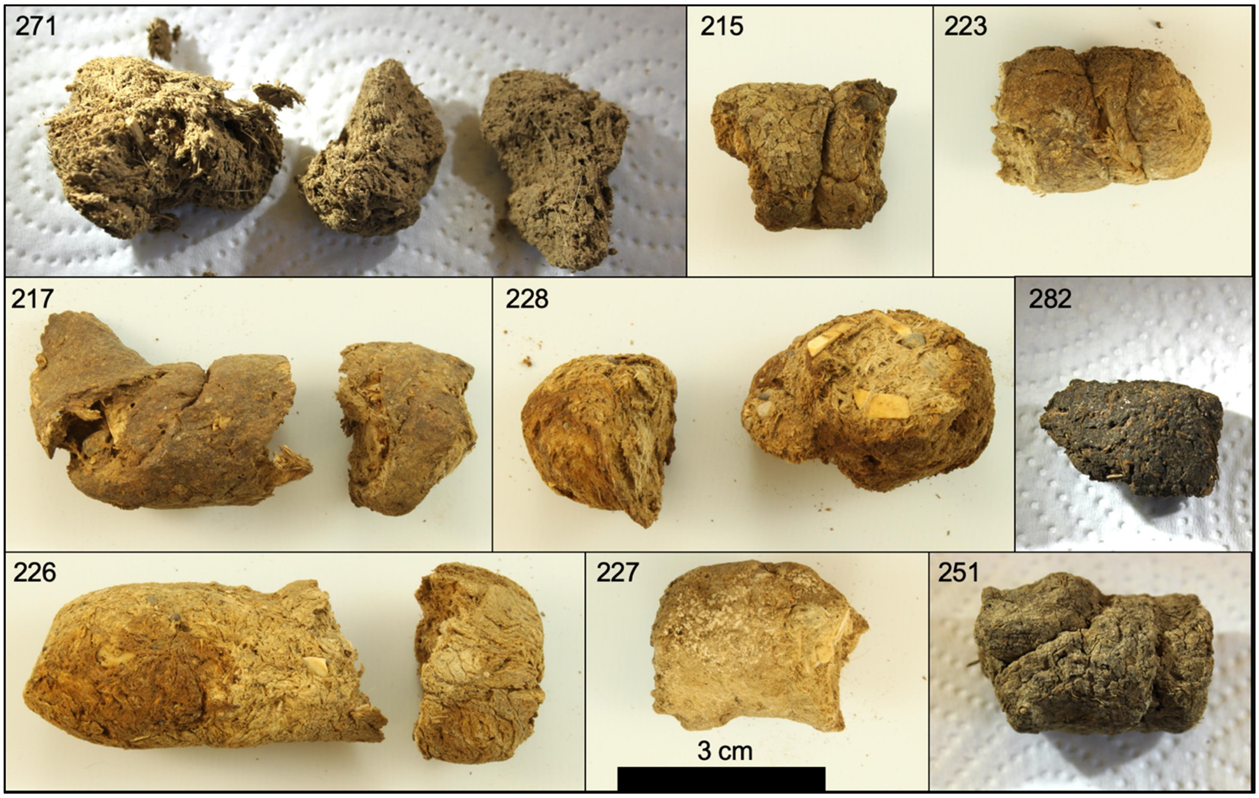

Fig. S5.

Figure S5, Images of Paisley coprolites prior to subsampling. Photo Credit: John Blong, Newcastle University.

Table S1.

Table S1. Comparative assessment of lipids derived from a sediment sample collected directly underneath coprolite 283.

Sample ID	Paisley catalogue number	Ratio 2 (*12*)	Ratio 3 (*12*)	Bile acids	Relative distributions
283C	1896-PC-5/16A-25-13a	0.5	0.5	None detected	Dominated by n-alcohols C_{22}-C_{32}. Coprostanol: 14.5%; Epicoprostanol: 2.7%; Cholesterol: 8.8%; 5α-Cholestanol: 16.1%; 5β-stigmastanol: 28.8%; Epi-5β-stigmastanol: 7.5%, 5α-stigmastanol: 21.6%.

Table S2.

Table S2. Radiocarbon date information for basket or mat fragment. [1] Calibrated using IntCal 2013 in Oxcal 4.3.

**Material dated**	**Paisley catalogue number**	**Lithic unit**	**^{14}C BP age**	**Lab number**	**Calibrated age cal B.P. (2σ)[1]**
Bulrush shaft with the features of an S-twist (clockwise) basket or mat weft fragment	1294-PC-5/7C-25-1	1b	12,273 ± 56	D-AMS-035352	14,545–14,003

Data S1. (separate file)

Context, morphological, metric, and previously published data for the Paisley caves coprolites presented in this study.
